# Validation of the three-level hepatectomy complexity classification and its AI application in robotic liver surgery

**DOI:** 10.1007/s13304-026-02724-5

**Published:** 2026-06-23

**Authors:** Alessandro D. Mazzotta, Francesca Ratti, Paolo Magistri, Andrea Belli, Graziano Ceccarelli, Francesco Izzo, Marcello Giuseppe Spampinato, Nicola de’ Angelis, Patrick Pessaux, Tullio Piardi, Fabrizio Di Benedetto, Michele Ammendola, Gianluca Mennini, Luca Aldrighetti, Michele Tedeschi, Riccardo Memeo

**Affiliations:** 1 Department of General, Specialist Surgery and Anestesiology, Sapienza University of Rome, 00185 Rome, Italy; 2 Hepatobiliary Surgery Division, IRCCS San Raffaele Scientific Institute, 20132 Milano, Italy; 3 Hepatobiliary Surgery Division, Vita-Salute San Raffaele University, 20132 Milano, Italy; 4 Unit of Hepato-Pancreato-Biliary Surgery and Liver Transplantation, University of Modena and Reggio Emilia, 41121 Modena, Italy; 5 Unit of Hepato-Biliary and Pancreatic Surgery, Istituto Nazionale Tumori IRCCS Fondazione G. Pascale, 80131 Naples, Italy; 6 Unit of General Surgery, San Giovanni Battista Hospital, USL Umbria 2, 06034 Foligno, Italy; 7 Department of General Surgery and sub-specialty, Asl Lecce, Department of Experimental Medicine, University of Salento, Lecce, 73100 Lecce, Italy; 8 Unit of Robotic and Minimally Invasive Digestive Surgery, Ferrara University Hospital, Ferrara, Italy; 9 Department of Visceral and Digestive Surgery, Unit of Hepato-Bilio-Pancreatic Surgery, Nouvel Hospital Civil, University Hospital of Strasbourg, 67000 Strasbourg, France; 10 Department of Hepatobiliary, Pancreatic and Digestive Oncological Surgery, Robert Debré University Hospital, Reims, France; 11 Department of Experimental and Clinical Medicine, Magna Graecia University Medical School, Digestive Surgery Unit, Renato Dulbecco University Hospital, Catanzaro, Italy; 12 Department of Hepato-Pancreatc-Biliary Surgery, “F. Miulli” General Regional Hospital, , Acquaviva Delle Fonti, Bari, Italy; 13 Department of Medicine and Surgery, LUM University, Casamassima, Bari, Italy

**Keywords:** Robotic liver surgery, Complication prediction, AI analysis, Surgical complexity, Machine learning, Postoperative outcomes

## Abstract

Robotic liver surgery (RLS) is expanding in recent years. Complication prediction is crucial for postoperative outcomes. Traditional MIS scores are poorly studied in RLS, and conventional statistics often oversimplify the multifactorial and interrelated nature of these complications. This study aimed to evaluate the three-level complexity Institut Mutualiste Montsouris (IMM) classification in RLS and assess its integration into an AI algorithm to predict major complications. We retrospectively analyzed data of patients underwent RLS. Surgical complexity was stratified into grades I (low complexity), II (intermediate), and III (high). The cumulative incidence rate and conditional probability of postoperative complication and risk factors for complication ≥ Clavien-Dindo grade II were assessed. The prediction model was developed by training/testing a machine learning (ML) algorithm after feature selection with uni-multivariate analysis. We calculated the receiver operating characteristic (ROC) curve and model accuracy. We analyzed 1,045 patients who underwent RLS, classifying them into three complexity levels: Grade I (n = 581), Grade II (n = 267), and Grade III (n = 109). Significant differences were observed in intra- and postoperative outcomes across the three grades. Multivariate analysis identified ASA score (HR 2.1, *p* = 0.02), number of lesions (HR 1.8, *p* = 0.001), and operative time (OR 1, *p* = 0.004) as key predictors of complications. Associated with the three-level complexity classification, the Neural Network showed the best performance with AUC (0.653) and a precision of 0.996. Three-level complexity IMM classification is a useful tool in RLS for predicting intra-postoperative outcomes. It can be integrated into the Neural Network algorithm to predict major complications.

**Supplementary Information:**

The online version contains supplementary material available at https://doi.org/10.1007/s13304-026-02724-5.

## Introduction

Laparoscopic liver resection (LLR) has been continuously gaining in popularity since it was first described in 1991 [1]. Since then, many surgeons and institutions have adopted minimally invasive laparoscopic techniques as the standard of care for liver resections. Randomized clinical trials have demonstrated the benefits of laparoscopic approach, which includes decreased intraoperative blood loss, lower risk of perioperative complications, and decreased mortality [2, 3].

Even if the LLR is safe and reproducible, it remains technically demanding, particularly for complex hepatectomies. Several studies have underlined the importance of surgeon’s experience, and the learning curve associated with application of laparoscopic approach to technically difficult resections [4, 5]. In this setting robotic surgery there has been a growing shift towards the robotic approach in recent years. Robotic liver surgery (RLR) should provide at least comparable benefits to open surgery, like those observed with LLR. Nevertheless, the evidence supporting the widespread adoption of RLR remains limited, and its costs are generally higher compared to laparoscopic surgery [6, 7].

A large study showed that RLR for the posterosuperior segments was associated with superior perioperative outcomes in terms of decreased operative time, blood loss, and open conversion rate when compared with LLR [8]. While both produce excellent outcomes, RLR might facilitate better outcomes than LLR [9].

Classification systems are important for planning liver resection procedures based on the learning curves of surgeons/teams and comparing intergroup differences of surgical complexity in cohort studies. They are well developed in LLR but a classification system analogous does not yet exist to guide surgeons undertaking robotic hepatectomies with varying technical difficulty levels.

The three-level complexity Institut Mutualiste Montsouris (IMM) classification system is one of several widely accepted difficulty scoring systems for LLR. It consists of a scale of grade I (low difficulty), grade II (intermediate difficulty), and grade III (high difficulty) based on the type/extent of liver resection. Grade I operations include wedge resection and left lateral sectionectomy, Grade II includes anterolateral segmentectomy and left hepatectomy and Grade III includes posterosuperior segmentectomy, right posterior segmentectomy, right hepatectomy, extended right hepatectomy, central hepatectomy, and extended left hepatectomy [10].

Recently, in addition to predictive difficulty scores which can sometimes seem very simplistic, it is useful to have an algorithm that can be both appropriate and efficient for predicting complications.

Artificial Intelligence (AI) is expanding its footprint in clinical systems, from databases to intraoperative video analysis. In the surgical setting, AI can assist in generating evidence-based, real-time clinical decision support designed to optimize patient care and surgeon workflow.

The use of AI enhances the ability of hepatobiliary surgeons to timely select the most suitable patients for complex surgical procedures, ensuring greater safety and personalized precision. Several studies have investigated different machine learning (ML) algorithms and artificial neural network (ANN) models to predict postoperative complications. AI has been utilized in this context, with Merath et al. [11] assessing an ML algorithm to predict the risk of complications following liver, pancreatic, or colorectal surgery. Another group of researchers focused on validating an ANN model to predict severe post-hepatectomy liver failure (PHLF) in patients with hepatocellular carcinoma (HCC) after hepatectomy [12].

Therefore, there is now a need to integrate AI algorithms into predictive models, alongside difficulty scores, to forecast postoperative outcomes following RLR.

The aim of our study was to assess the three-level complexity classification as a tool for intra and postoperative outcomes in RLR. The second aim was to evaluate this three-level complexity classification alongside other variables within a ML algorithm to assess its ability to prevent major complications.

## Patients and methods

### Study cohort

This was a retrospective multicentric study based on a clinical institutional database.

The Independent ethics committee approved the protocol of the study. We analyzed patients undergoing RLR for malignant and non-malignant liver disease from December 2011 to May 2023.

Surgical procedures were performed using Da Vinci Si from 2011 to 2017 and with the Da Vinci Xi from 2018 to 2023.

All procedures were performed by experienced hepatobiliary surgeons with established expertise in minimally invasive and robotic liver surgery. Although the operating surgeon was not the same across all cases, all participating surgeons had completed their learning curve prior to inclusion in the study.

The inclusion criteria were adults aged ≥ 18 years, with a diagnosis of liver tumors (either primary or metastatic) requiring surgical intervention. Patient demographic and clinical data were collected from electronic medical records and included variables such as age, gender, body mass index (BMI), comorbidities (e.g., diabetes, cardiovascular disease), preoperative liver function tests, tumor characteristics (size, location, number), and type of liver resection (minor, major, or extended hepatectomy). Intraoperative details such as duration of surgery, estimated blood loss, type of resection, and the use of vascular clamping were also documented.

Informed consent was obtained from all patients or their legal representatives prior to data collection.

### Preoperative management

The final decision for surgical intervention was discussed on a case-by-case basis in a multidisciplinary meeting.

The future liver remnant (FLR) was assessed through a liver volumetry calculated on a three-dimensional computed tomography scan (CT). When the FLR was considered insufficient, a portal vein embolization was performed. Patients were placed in a low lithotomy position, with their legs split and knees bent (French position). Parenchymal transection was performed using the clamp-crush method and/or laparoscopic ultrasonic dissector, performed by patient side surgical assistant, with or without a Pringle’s maneuver. Drains were only placed if there was concern about a risk of postoperative bleeding and bile leakage.

### Definitions

The operative time of the surgical procedure was defined as the time from the first skin incision to the final skin closure. Estimated blood loss (EBL) was based on the anesthesia records at the end of the operation. Surgical complications were defined as any deviation from the normal postoperative course within 90 days after liver resection. Complications were graded according to the Clavien-Dindo (C-D) classification and complication ≥ 3a was defined as a major complication [13].

Hepatectomies were classified using the three-level complexity classification already published: grade I (wedge resection for anterolateral and posterosuperior segment and left lateral sectionectomy), grade II (segmentectomy of anterolateral segment and left hepatectomy), and grade III (posterosuperior segmentectomy, right posterior sectionectomy, right hepatectomy, central hepatectomy and extended left/right hepatectomy) [10]. See supplementary Fig. 1 [14].

### Statistical analysis

Quantitative data are expressed as the means with standard deviations or medians with ranges. Qualitative data are expressed as numbers with percentages. The Kruskal–Wallis test was used to compare continuous variables between groups. Pearson’s chi-square or Fisher’s exact test was used to compare categorical variables as appropriate. Factors significantly associated with Major complication were identified using univariable and multivariable Cox analyses.

The significance level to allow the inclusion of a variable in the logistic multivariate regression model was 0.05.

### Machine learning methodology

The ML model development and validation were conducted using the *Orange ® data mining software*. We selected the most important variables after a uni-multivariate analysis; subsequently, we associated these variables with the three-level complexity classification, considering it as input. The purpose was to support the three-level complexity classification with additional variables in AI algorithms for the prediction of major complications, which was considered the outcome of interest.

Four different ML algorithms were examined: Logistic Regression, Random Forest, ANN, and k-Nearest Neighbors (kNN).

The ANN model consists of an input layer, multiple hidden layers, and an output layer. The hidden layers utilized the ReLU (Rectified Linear Unit) activation function, while the output layer applied a sigmoid function to classify major complications. The random forest method is multiple decision trees during training and determined the final classification based on a majority vote. k-Nearest Neighbors (kNN) is a non-parametric algorithm classified patients based on the majority vote of their k nearest neighbors. Logistic Regression is a model that use to predict major complications by analyzing relationships between patient features and outcomes. To prevent overfitting, it included L2 regularization (Ridge Regression).

#### Data preprocessing

To guarantee that every feature contributed equally to the model’s performance, the data was pre-processed prior to model training to accommodate missing values and normalize the numerical variables. We convert categorical variables into a format that was appropriate for the algorithms, they were also one-hot encoded.

#### Model training and evaluation

We divided the dataset into training and test sets using a 70/30 split, 70% of the data allocated for model training and 30% for testing. The training and assessment procedure was carried out more than 100 times to guarantee the models’ resilience and dependability.

#### Performance metrics

Model performance was assessed using the Area Under the Curve (AUC) of the Receiver Operating Characteristic (ROC) curve. Furthermore, the Matthews Correlation Coefficient (MCC) was employed to provide a more balanced evaluation. The MCC metric accounts for true and false positives and negatives, delivering a single score that reflects the quality of predictions across all classes.

#### Model selection

After iteratively training and evaluating the models, their performances were compared. The algorithm that exhibited the highest average AUC and MCC scores across all iterations was selected as the optimal model for predicting major complication. The final model was then validated on the test set to confirm its predictive accuracy and generalizability.

#### Cross-validation methodology

To further ensure robustness and minimize the impact of overfitting, Stratified 20-fold Cross Validation was applied. This method ensures that the distribution of classes is preserved within each fold, providing a more balanced evaluation of the models. A total of 957 instances with 5 features (3 categorical and 2 numeric) were used for the evaluation. The dataset had 1.2% missing values, which were appropriately handled during preprocessing.

## Results

A total of 1,071 patients underwent robotic liver resections, with 1,045 included in the study. Among these 957 patients underwent a single robotic liver resection. Notably, 27 patients were excluded due to missing data concerning the surgical procedure, and 87 underwent multiple resections.

**Fig. 1:**
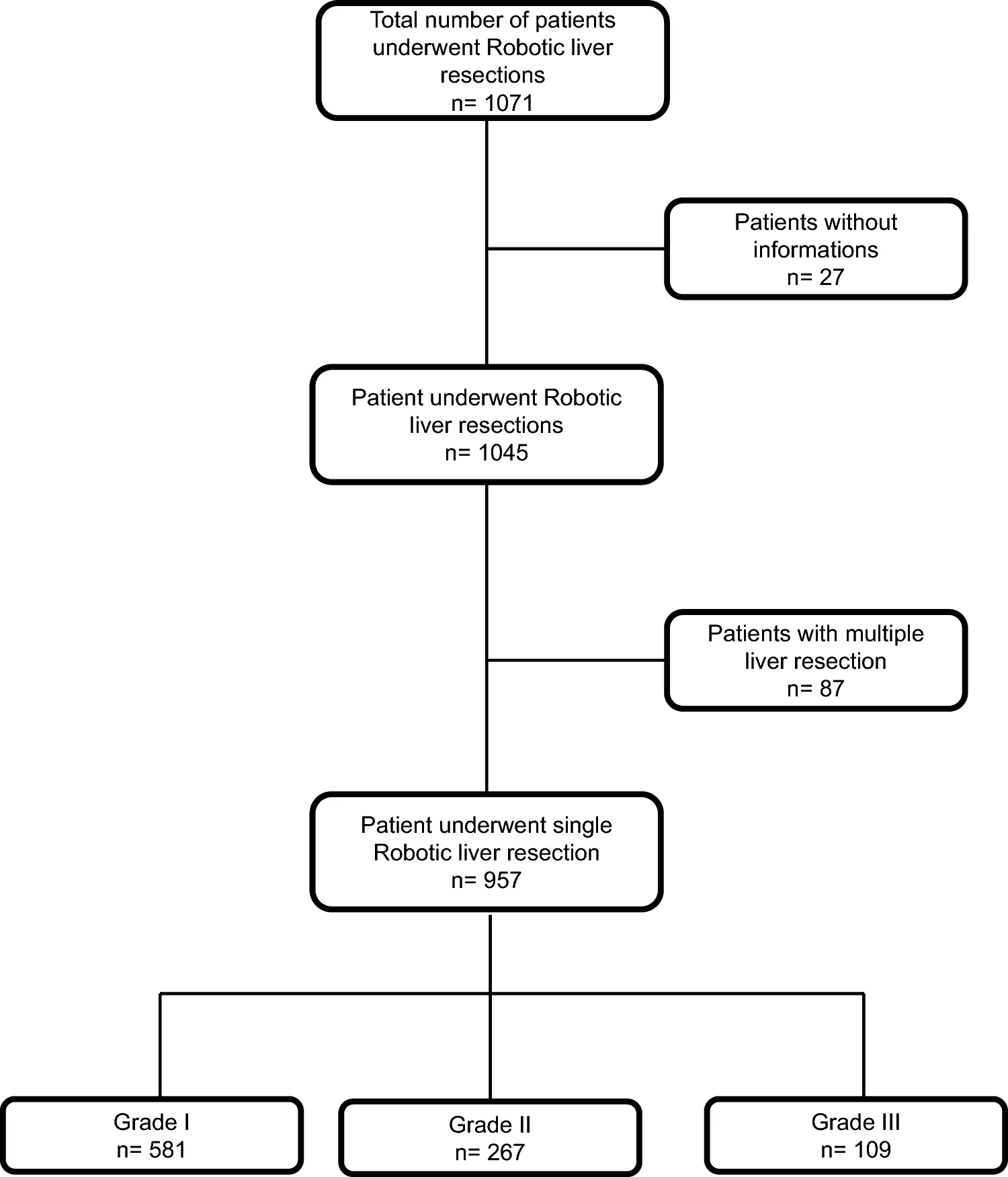
Study flowchart showing patient selection and stratification according to the three-level complexity classification

We classified patients who underwent a single resection according to the three-level complexity classification: Grade I (n = 581), Grade II (n = 267), and Grade III (n = 109). (Fig. 1).

### Preoperative characteristics

Analysis of preoperative characteristics revealed no significant differences in age, sex, BMI, or ASA classification among the three grades.

However, significant differences were observed for cirrhosis, with Grade II patients showing a higher prevalence (43.1%) compared to Grade I (32.7%) and Grade III (25.9%) (*p* = 0.002). Additionally, the number of lesions and tumor size were significantly larger in Grade III patients, with median tumor sizes of 39 mm compared to 25 mm in Grade I and 32 mm in Grade II (*p* = 0.001). (Table 1).

**Table 1:** Preoperative characteristic of the groups according the IMM 3 complexity grade

	Total n = 957	Grade I = 581	Grade II = 267	Grade III = 109	*p* value
Age	66 [57–73]	65 [57–73]	68 [59–74]	64 [55–73]	0.139
Sex M	565 (59.2)	334 (57.8)	163 (61)	68 (62.4)	0.518
BMI	26 [24–28]	26 [24–28]	26 [24–28]	26 [25–29]	0.140
ASA					0.472
1	44 (4.7)	26 (4.5)	11 (4.2)	7 (6.5)	
2	496 (52.5)	293 (51)	138 (52.5)	65 (60.2)	
3	404 (42.8)	254 (44.3)	114 (43.3)	36 (33.3)	
4	1 (0.1)	1 (0.2)	0 (0)	0 (0)	
Cirrhosis	327 (34.9)	286 (32.7)	113 (43.1)	28 (25.9)	0.002
Previous Surgery	452 (47.2)	300 (51.6)	108 (40.4)	44 (40.4)	0.003
Number of lesion	1 [1, 2]	1 [1–1]	1 [1–1]	1 [1, 2]	0.001
Tumor size	30 [20–43]	25 [18–38]	32 [22–50]	39 [30–50]	0.001

() = %; [] = IQR

### Intraoperative and postoperative outcomes

Median estimated blood loss (EBL) increased progressively with complexity, being highest in Grade III patients (EBL of 265 mL), compared to 100 mL in Grade I and 150 mL in Grade II (*p* = 0.001). Median Operative time was also significantly longer in Grade III (300 min) compared to Grade I (200 min) and Grade II (220 min) (*p* = 0.001). Clamping time followed a similar pattern, with Grade III requiring a median of 40 min, significantly longer than the 0 min in Grade I and minimal clamping in Grade II (*p* = 0.001).

The conversion rate to open surgery was notably higher in Grade III patients (12.1%) compared to Grade I (4%) and Grade II (7.8%) (*p* = 0.001). However, no significant differences were observed in the length of hospital stay or the overall rate of general complications (*p* > 0.05). Major complications were more frequent in Grade III patients (10.3%) compared to Grade I (4.6%) and Grade II (6.3%), with a p-value of 0.05.

Mortality rates were observed to increase with the level of complexity: 0% for Grade I, 0.4% for Grade II, and 1.8% for Grade III. While there is a noticeable trend toward higher mortality in more complex cases, the difference did not reach statistical significance (*p* = 0.07). (Table 2).

**Table 2:** Intraoperative and post operative results

	Total n = 957	Grade I = 581	Grade II = 267	Grade III = 109	*p* value
EBL	150 [150–300]	100 [50–200]	150 [100–340]	265 [157–400]	0.001
Operative time (min)	210 [160–300]	200 [150–270]	220 [180–320]	300 [240–370]	0.001
Clamping (min)	0 [0–30]	0 [0–15]	0 [0–35]	40 [0–65]	0.001
Hospital length stay (days)	5 [4–6]	5 [4–6]	5 [4–7]	5 [4–7]	0.35
Conversion to open	57 (6)	23 (4)	21 (7.8)	13 (12.1)	0.001
General Complication	169 (17.6)	88 (15.1)	60 (22.3)	21 (19.6)	0.10
Major complication	55 (5.7)	27 (4.6)	17 (6.3)	11 (10.3)	0.05
R1 rate	44 (4.5)	26 (4.5)	10 (3.7)	8 (7.5)	0.25
Readmission	43 (4.4)	26 (4.5)	12 (4.5)	5 (4.7)	0.002
Mortality	3 (0.3)	0 (0)	1 (0.4)	2 (1.8)	0.07

() = %; [] = IQR; EBL = estimated blood loss

### Predictors of major complications

Considering the three-level complexity classification as a single tool for major complications prediction the AUC is 0.57.

Univariate analysis identified age over 65 years, ASA score, the number of lesions, operative time, and EBL as significant predictors of major complications. Multivariate analysis confirmed ASA score (HR = 2.1, *p* = 0.02), the number of lesions (HR = 1.8, *p* = 0.001), and operative time (HR = 1.08, *p* = 0.004) as independent predictors of major complications. (Table 3).

**Table 3:** Univariate and multivariate analysis for Major complication

	Univariate analysis			Multivariate analysis		
	HR	CI	*P* value	HR	CI	*P* value
Age > 65	1.8	1.00–3.25	0.050	1.70	0.85–3.39	0.12
BMI	1.00	0.92–1.06	0.850			
ASA	1.7	1.07–2.97	0.025	2.1	1.10–4.0	0.02
Previous Surgery	1.26	0.73–2.17	0.401			
Cirrhosis	1.05	0.57–1.18	0.960			
Number of lesion	1.48	1.18–1.85	0.001	1.8	1.39–2.56	0.001
Tumor size	1.00	0.94–1.01	0.400			
Clamping	0.70	0.99–2.19	0.050	1.34	0.60–2.70	0.40
Operative time	1.00	1.00–1.00	0.001	1	1.00–1.08	0.004
EBL	1.00	1.00–1.02	0.004	1	0.99–1.00	0.46

### AI algorithm evaluation with the three-level complexity classification and other variables

**Fig. 2:**
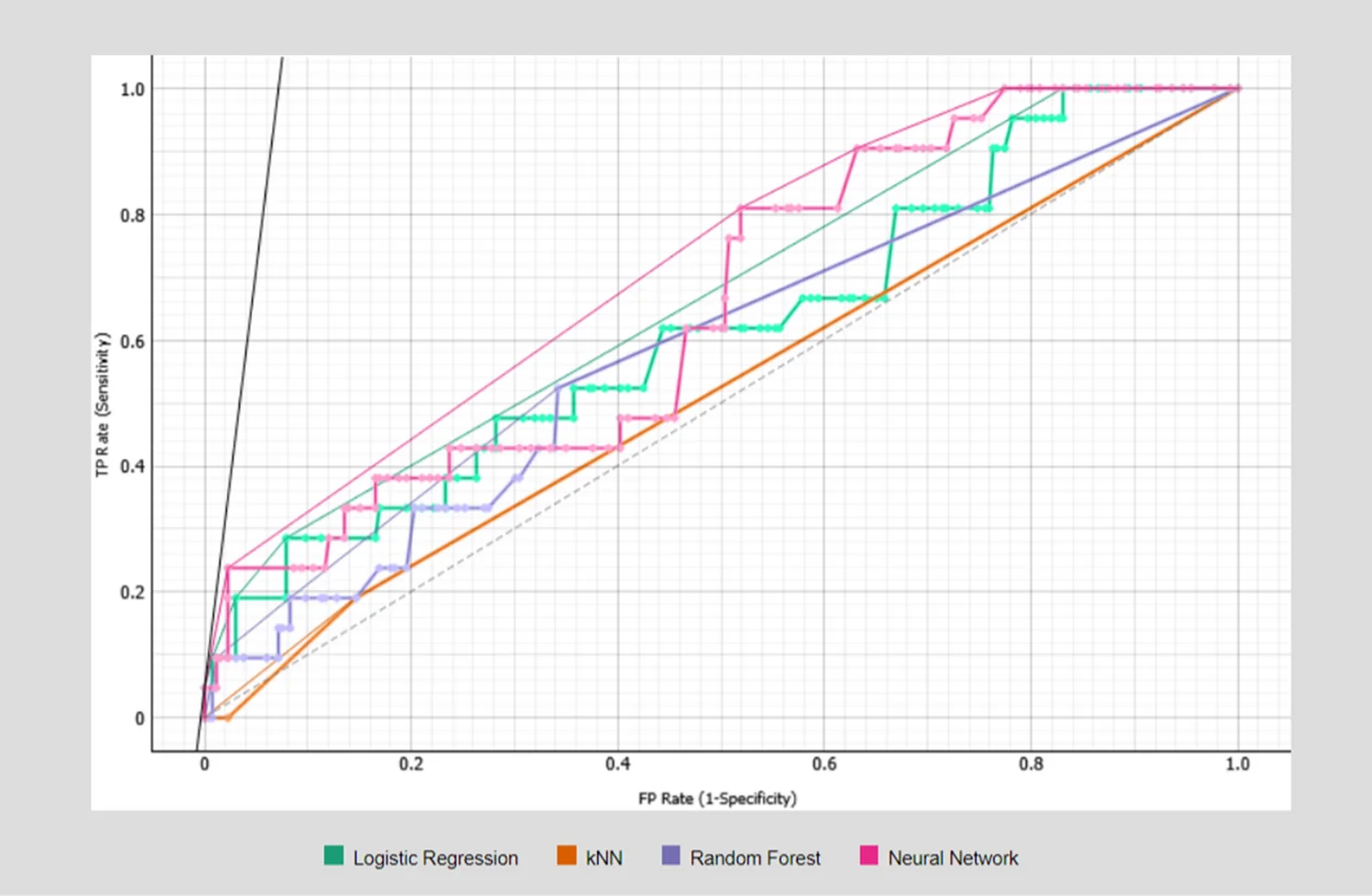
ROC curves comparing the performance of machine learning models for predicting major postoperative complications

The ANN model achieved an AUC of 0.653, a classification accuracy of 0.927, an F1 score of 0.962, precision of 0.930, recall of 0.996, and an MCC of 0.137. The Logistic Regression model resulted in an AUC of 0.615, a classification accuracy of 0.930, an F1 score of 0.964, precision of 0.930, recall of 1.000, and an MCC of 0.210. (Table 4 and Fig. 2).

**Table 4:** Comparison of ML algorithm

Model	AUC	CA	F1	Prec	Recall	MCC
Neural Network	0.653	0.927	0.962	0.930	0.996	0.137
Logistic Regression	0.615	0.930	0.964	0.930	1.000	0.210
Random Forest	0.555	0.923	0.960	0.930	0.992	0.103
kNN	0.520	0.927	0.962	0.927	1.000	0.000

The results of cross validation (supplementary Table 1) confirmed ANN model as the best model. The ANN model achieved an AUC of 0.628, a classification accuracy of 0.945, an F1 score of 0.921, precision of 0.934, recall of 0.945, and an MCC of 0.193.

## Discussion

To our knowledge this is the first study comparing the three-level complexity classification score in a multicentric study in RLR and integrating it into a ML algorithm. Our vision was that the three-level complexity classification score, while effective as a foundational predictive tool, lacks the depth to fully capture the multifaceted nature of patient conditions and surgical complexity. This underscores the need for AI-driven solutions to enhance its predictive capabilities and provide a more comprehensive risk assessment framework.

Concerning our results, patient demographics such as age, sex, and BMI were similar across different complexity grades of liver resections, with no significant differences observed, but cirrhosis was significantly more prevalent in Grade II patients.

As evidenced by our results, Grade I hepatectomies are more common compared to Grades II and III. This is partly because Grade III resections are rarer, but also because many centers likely preferred to use the robotic approach for Grade I cases, possibly as part of a stabilization phase during the learning curve. This study does not aim to evaluate the efficiency of robotic surgery in Grade I cases, which can typically be approached laparoscopically, reducing costs without necessarily requiring robotic surgery. Our objective was to retrospectively describe the cases and associate them with predictive outcomes relative to the levels of complexity.

In comparison to the study conducted by Sucandy et al. [15] the three-level complexity classification is a strong predictor of intraoperative outcomes, such as estimated blood loss (EBL) and operative time. We confirmed these results in our study and in particular in the current dataset, a significant increase in EBL and operative time was observed with increasing three-level complexity classification. For instance, the EBL increases progressively from 100 ml in Grade I to 265 ml in Grade III in the present study, while Sucandy et al. report a similar trend with significant differences across the grades (*p* < 0.001 for both outcomes). While both studies found significant differences in conversion to open surgery rates, the rates were higher in the present study (6% overall) compared to Sucandy et al. (4.5%). This disparity might be the result of variations in patient characteristics, or surgical skill, likely due to the multicentric nature of the study.

Although mortality rates were observed to increase with higher complexity levels, reaching 1.8% in Grade III resections compared to 0% in Grade I and 0.4% in Grade II, this difference did not achieve statistical significance (*p* = 0.07). Despite this trend, our findings should not be interpreted as discouraging the use of robotic approaches for Grade III hepatectomies. The robotic platform offers significant advantages, particularly in precision, visualization, and instrument dexterity, which may be critical for managing such complex cases.

The use of evidence-based practices has significantly replaced anecdotal knowledge and firsthand experience in clinical practice in recent years. This shift is a result of the growing accessibility of thorough clinical data as well as the use of advanced data analysis methods like ML. A key field in this development is surgical data science (SDS), which combines traditional clinical approaches with ML algorithms to improve patient outcomes. Large-scale datasets may be analyzed with SDS, yielding fresh perspectives and instruments for decision-making that enhance preoperative preparation, intraoperative efficiency, and postoperative results. [16].

Compared to conventional statistical techniques, the application of ML in this study offers several clear benefits. The capacity to incorporate variables with partial data—which are frequently left out of traditional analyses—is one of the main advantages; nevertheless, in our analysis, there were not many missing data points.

The three-level complexity classification score is a preoperative predictive tool that has already been validated; however, it remains somewhat simplistic at times and does not account for certain important variables that a patient may present. Surgical indications are increasingly being extended to more compromised patients, making it essential to have a comprehensive view of the patient. In this context, AI can be of great help, providing surgeons with a highly valuable tool.

A critical aspect of our model concerns the nature of the included variables. While ASA score and number of lesions represent preoperative parameters, operative time is inherently an intraoperative variable. This introduces a limitation in terms of traditional clinical applicability, as most established risk stratification systems in liver surgery are designed to provide purely preoperative predictions to guide patient selection and informed consent.

However, this apparent limitation may also reflect a paradigm shift toward dynamic and precision surgery. In complex procedures such as robotic liver resection, intraoperative factors play a pivotal role in determining outcomes, and unexpected events frequently occur despite accurate preoperative planning. In this context, incorporating intraoperative variables may enhance the model’s ability to capture real-world surgical complexity and support adaptive, real-time risk assessment.

Therefore, rather than viewing this as a methodological weakness alone, our approach suggests a complementary strategy, where predictive models evolve from static preoperative tools to dynamic systems integrating intraoperative data to guide surgical decision-making and improve precision surgery.

Our approach was to evaluate the predictive ability of the three-level complexity classification score alongside other important variables within a machine learning algorithm. In other words, the AUC increases from 0.57 when considering the three-level complexity classification score alone to 0.65 when incorporating additional variables selected in the ANN algorithm. The IWATE score, although it already integrates additional information such as proximity to major vessels, liver pathology, etc., [17] has not been utilized in the present study. This could be attributed to its complexity and the focus of this study on the simplicity of the three-level complexity classification, which requires integration with other variables. The IWATE score will be the subject of future research efforts.

We tested four different algorithms—ANN, Logistic Regression, Random Forest, and k-Nearest Neighbors (kNN)—to assess their effectiveness in predicting major complications. Each model was evaluated using a variety of performance metrics, including AUC, classification accuracy, F1 score, precision, recall, and Matthews Correlation Coefficient (MCC).

In our evaluation of the four ML models, the ANN and Random Forest models demonstrated the best overall performance, with AUCs of 0.645 and 0.643, respectively. Although these AUC values indicate moderate predictive power, the high classification accuracy and F1 scores suggest that these models are effective in correctly classifying patients at risk of major complications.

However, the relatively low MCC values across all models (ranging from 0.017 for kNN to 0.156 for the ANN) indicate that while the models perform well in terms of classification accuracy, they may not be as reliable when accounting for the balance between true and false positives and negatives. This suggests that while ML models can be useful for predicting complications, their results should be interpreted with caution, especially in clinical contexts where the cost of false predictions is high. This caution is particularly crucial in RLR procedures, where unanticipated complications can significantly impact patient outcomes.

Other studies have also used neural networks such as this study by Bondie et al. [18] that used deep neural networks (DNN) to simulate a variety of preoperative and intraoperative parameters with the purpose of predicting postoperative problems, with an AUC of 0.874. However, Bonde’s study uses neural networks to predict numerous problems concurrently (multilabel), enhancing the overall accuracy.

This study primarily evaluated the AUC as the principal parameter for assessing the prediction ability of several ML models regarding surgical complications. The AUC is extensively utilized in classification models, particularly in medical research, as it quantifies a model’s ability to differentiate between two classes at various thresholds, rendering it less susceptible to class imbalance than accuracy alone. Our study revealed that the models produced AUC values between 0.520 and 0.653, with the Neural Network model attaining the highest AUC of 0.653. This number indicates intermediate discriminative capability, suggesting potential for enhancement. It is important to note that although F1 scores and accuracy (CA) were elevated across models, these measures may occasionally result in erroneous interpretations in unbalanced datasets, as emphasized by Chicco et al. [19].

Although the ANN model demonstrated the best performance among the tested algorithms, the overall discriminative ability remained moderate, with an AUC of approximately 0.65. From a clinical standpoint, this level of accuracy does not support the use of the model as a standalone decision-making tool. Rather, it should be interpreted as an adjunct to clinical judgment, providing probabilistic support rather than definitive risk stratification.

This limitation is particularly relevant in minimally invasive hepatobiliary surgery, where decision-making relies on a complex integration of anatomical, oncological, and patient-related factors that may not be fully captured by the current dataset. Therefore, while the model highlights meaningful associations, its clinical applicability remains restricted, and further refinement with larger datasets and additional variables is required before routine implementation.

In another study, the authors utilized MCC in conjunction with AUC to evaluate their prediction model for postoperative complications in thoracic surgery. Their methodology revealed that MCC might uncover inconsistencies not identified by accuracy or F1 score alone, especially in the prediction of infrequent yet significant problems [16]. In our analysis, MCC values were predominantly low across models, with the highest MCC observed at 0.210 for Logistic Regression.

Concerning the future prospective we would like to assess the applicability of these models, which have previously been demonstrated to show significant correlations when utilizing univariate and multivariate models. If this works, it might increase the accuracy of predicting different surgical problems. In RLR, where visual and motor accuracy are crucial, improved predictive models may facilitate real-time decision-making, thus reducing operating risk. The transition to ML-based models requires strict data and variable management to avoid overfitting and provide robustness across different patient cohorts.

This study represents a preliminary effort not only to utilize conventional classification metrics and analyse variables statistically but also to introduce a novel approach where traditional statistical analysis alone is insufficient. We aimed to emphasize the importance of developing integrated systems that combine statistical methods with advanced machine learning techniques to enhance predictive power. Given the challenges and complications associated with minimally invasive liver surgery, there is a necessity to advance beyond conventional statistical models. Our methodology aims to tackle the intricate and multifactorial aspects of surgical risk prediction in RLR, highlighting the requirement for advanced, hybrid solutions to enhance predictive accuracy and facilitate clinical decision-making in more nuanced manners.

This study should be interpreted in light of several limitations. The retrospective design of our investigation involves possible biases, especially regarding data collection and patient selection. Despite employing a multicentric dataset, the variability in institutional protocols and surgeon expertise may have affected the results. This diversity may not be sufficiently represented by the ML models, thus constraining their capacity to generalize across diverse contexts. The absence of defined surgical methodologies may lead to inconsistencies in procedural complexity.

Secondly, the sample size, while substantial, may restrict the generalizability of the produced machine learning models. The dataset, although typical, does not include all possible variables that may affect surgical outcomes, such as long-term follow-up data or comprehensive patient comorbidities. The lack of an external validation set hinders a comprehensive evaluation of model robustness and generalizability in practical applications, creating uncertainty over the models’ performance with patient data from diverse institutions with differing characteristics. Moreover, the models’ limited predictive capability—particularly the low MCC values—suggests that these algorithms necessitate enhancement and validation to optimize their clinical significance. The low MCC indicates a restricted ability to balance genuine positives, true negatives, and incorrect predictions, hence raising worries over their trustworthiness in clinical decision-making, where prediction errors may have serious implications.

Another important limitation concerns the heterogeneity of baseline preoperative characteristics across the three complexity groups. In particular, significant differences were observed in liver cirrhosis, number of lesions, and tumor size. For instance, cirrhosis was more prevalent in Grade II patients, whereas Grade III patients—despite representing the highest surgical complexity—were paradoxically less cirrhotic but presented with larger tumors and a higher burden of disease. The three-level IMM classification is primarily based on technical difficulty of the resection rather than biological disease severity. As a consequence, higher complexity procedures (Grade III) may not necessarily correspond to more compromised patients, but rather to more technically demanding resections. Importantly, when these variables were tested in the univariate and multivariate analyses, most of them—including cirrhosis and tumor size—were not independently associated with major complications, with the exception of the number of lesions, which remained a significant predictor. This suggests that, despite baseline heterogeneity, the impact of these variables on postoperative outcomes may be less pronounced than expected when adjusted within a multivariable framework.

A potential limitation concerns the lack of explicit reporting of tumor location. These variables are inherently incorporated within the three-level IMM classification, which is primarily based on technical surgical complexity. However, in a multicentric setting, differences in tumor location may introduce a degree of heterogeneity and potential misclassification across centers.

Another limitation relates to the long study period and the multicentric nature of the dataset. Although all procedures were performed by experienced hepatobiliary surgeons, variability in surgical teams and institutional practices may have influenced outcomes. In addition, two different robotic platforms (da Vinci Si and Xi) were used during the study period. While the Xi system introduced technical improvements in terms of docking, instrument reach, and multi-quadrant access, the fundamental principles of robotic liver dissection—including the absence of true haptic feedback and the reliance on visual cues—remained consistent across platforms. Therefore, although a major conceptual impact on surgical technique is unlikely, minor technical differences and progressive technological evolution may have contributed to heterogeneity. Furthermore, the potential influence of learning curve dynamics across centers cannot be entirely excluded.

Ultimately, our study does not explicitly examine the ethical ramifications of integrating AI in healthcare, encompassing data privacy issues, algorithmic biases, and the potential effects on clinical decision-making. These ethical considerations are crucial for ensuring responsible AI development, particularly in the management of sensitive patient data.

## Conclusions

In this study, we demonstrated the applicability and utility of the three-level IMM complexity classification for robotic liver resections. However, the three-level IMM complexity classification system alone has limitations in capturing the full spectrum of patient and procedural complexity, highlighting the need for AI support. The integration of the three-level complexity classification into ML algorithms offers an innovative approach to enhancing preoperative planning and patient management, with the potential to improve surgical outcomes. Among the tested models, ANN emerged as the most effective ML algorithm for this purpose.

## Supplementary Information

Below is the link to the electronic supplementary material.

Supplementary file1 (DOCX 81 KB)

## Data Availability

The datasets used and analyzed during the current study are available from the corresponding author on reasonable request.

## References

[1] Laparoscopic excision of benign liver lesions - PubMed [Internet]. [cited 2024 Aug 10]. Available from: https://pubmed.ncbi.nlm.nih.gov/1833688/

[2] Chiow AKH, Fuks D, Choi GH, Syn N, Sucandy I, Marino MV et al (2021) International multicentre propensity score-matched analysis comparing robotic versus laparoscopic right posterior sectionectomy. Br J Surg 108(12):1513–1520 10.1093/bjs/znab321 PMC8743054 34750608

[3] Yoshida H, Taniai N, Yoshioka M, Hirakata A, Kawano Y, Shimizu T et al (2019) Current status of laparoscopic hepatectomy. J Nippon Med Sch 86(4):201–206 10.1272/jnms.JNMS.2019_86-411 31204380

[4] Lin NC, Nitta H, Wakabayashi G (2013) Laparoscopic major hepatectomy: a systematic literature review and comparison of 3 techniques. Ann Surg 257(2):205–213 10.1097/SLA.0b013e31827da7fe 23263192

[5] Kluger MD, Vigano L, Barroso R, Cherqui D (2013) The learning curve in laparoscopic major liver resection. J Hepatobiliary Pancreat Sci 20(2):131–136 10.1007/s00534-012-0571-1 23064988

[6] Görgec B, Zwart M, Nota CL, Bijlstra OD, Bosscha K, De Boer MT, et al. (2023) Implementation and outcome of robotic liver surgery in The Netherlands: a nationwide analysis. Ann Surg [Internet]. 277(6):E1269–77. Available from: https://pubmed.ncbi.nlm.nih.gov/35848742/10.1097/SLA.0000000000005600 PMC10174096 35848742

[7] Hobeika C, Pfister M, Geller D, Tsung A, Chan A, Troisi RI, et al.(2024) Recommendations on robotic Hepato-Pancreato-biliary surgery. The Paris Jury-Based Consensus Conference. Ann Surg [Internet]. [cited 2024 Oct 29]; Available from: https://journals.lww.com/annalsofsurgery/fulltext/9900/recommendations_on_robotic.901.aspx 10.1097/SLA.0000000000006365 38787528

[8] Krenzien F, Schmelzle M, Pratschke J, Feldbrügge L, Liu R, Liu Q, et al. (2024) Propensity score-matching analysis comparing robotic versus laparoscopic limited liver resections of the Posterosuperior segments: an international multicenter study. Ann Surg [Internet]. 2024 Feb 1 [cited 2024 Aug 10];279(2):297–305. Available from: https://pubmed.ncbi.nlm.nih.gov/37485989/10.1097/SLA.0000000000006027 37485989

[9] Sijberden JP, Hoogteijling TJ, Aghayan D, Ratti F, Tan EK, Morrison-Jones V, et al. (2024) Robotic versus laparoscopic liver resection in various settings: an international multicenter propensity score matched study of 10.075 Patients. Ann Surg [Internet]. 280(1):108. Available from: /pmc/articles/PMC11161239/10.1097/SLA.0000000000006267 PMC11161239 38482665

[10] Kawaguchi Y, Fuks D, Kokudo N, Gayet B (2018) Difficulty of laparoscopic liver resection. Ann Surg 267(1):13–17 10.1097/SLA.0000000000002176 28187043

[11] Merath K, Hyer JM, Mehta R, Farooq A, Bagante F, Sahara K, et al. (2020) Use of machine learning for prediction of patient risk of postoperative complications after liver, pancreatic, and colorectal surgery. J Gastrointestinal Surgery. 2020;24(8). 10.1007/s11605-019-04338-2 31385172

[12] Mai R yun, Lu H ze, Bai T, Liang R, Lin Y, Ma L, et al. (2020) Artificial neural network model for preoperative prediction of severe liver failure after hemihepatectomy in patients with hepatocellular carcinoma. Surgery (United States). 168(4). 10.1016/j.surg.2020.06.031 32792098

[13] Dindo D, Demartines N, Clavien PA, Clavien PA. (2004) Classification of surgical complications a new proposal with evaluation in a cohort of 6336 patients and results of a survey. Annals Surg. 240(2). 10.1097/01.sla.0000133083.54934.ae PMC1360123 15273542

[14] Kawaguchi Y, Lillemoe HA, Vauthey JN. Surgical Resection: Old Dog, Any New Tricks? Vol. 24, Clinics in Liver Disease. 2020. 10.1016/j.cld.2020.07.004 33012450

[15] Sucandy I, Kang RD, Adorno J, Crespo K, Syblis C, Ross S et al (2023) Validity of the Institut Mutualiste Montsouris classification system for robotic liver resection. HPB 25(9):1022–1029 10.1016/j.hpb.2023.05.004 37217370

[16] Salati M, Migliorelli L, Moccia S, Andolfi M, Roncon A, Guiducci GM, et al. (2021) A machine learning approach for postoperative outcome prediction: surgical data science application in a thoracic surgery setting. World J Surg 45(5):1585–94. Available from: https://pubmed.ncbi.nlm.nih.gov/33594578/10.1007/s00268-020-05948-7 33594578

[17] Wakabayashi G (2014) What has changed after the Morioka consensus conference 2014 on laparoscopic liver resection? Hepatobiliary Surg Nutr. 10.21037/hbsn.2016.03.03 PMC4960417 27500140

[18] Bonde A, Varadarajan KM, Bonde N, Troelsen A, Muratoglu OK, Malchau H et al (2021) Assessing the utility of deep neural networks in predicting postoperative surgical complications: a retrospective study. Lancet Digit Health. 10.1016/s2589-7500(21)00084-4 34215564

[19] Chicco D, Jurman G (2020) The advantages of the Matthews correlation coefficient (MCC) over F1 score and accuracy in binary classification evaluation. BMC Genom. 10.1186/s12864-019-6413-7 PMC6941312 31898477

