# Supplementary material for: Validation of the three-level hepatectomy complexity classification and its AI application in robotic liver surgery
Source: Updates Surg. 2026 Jun 23;78(6):2389–99. doi: 10.1007/s13304-026-02724-5 (PMC13736433; doi:10.1007/s13304-026-02724-5)
Supplement: Supplementary file 1 [file 13304_2026_2724_MOESM1_ESM.docx]

Supplementary table 1. Cross Validation

| Model | AUC | CA | F1 | Precision | Recall | MCC |
| --- | --- | --- | --- | --- | --- | --- |
| Neural Network | 0.628 | 0.945 | 0.921 | 0.934 | 0.945 | 0.193 |
| Random Forest | 0.618 | 0.936 | 0.917 | 0.905 | 0.936 | 0.093 |
| Logistic Regression | 0.618 | 0.944 | 0.917 | 0.947 | 0.944 | 0.131 |
| k-Nearest Neighbors (kNN) | 0.581 | 0.940 | 0.914 | 0.888 | 0.940 | -0.011 |
